# Examination of the Relationship between Oral Health and Arterial Sclerosis without Genetic Confounding through the Study of Older Japanese Twins

**DOI:** 10.1371/journal.pone.0127642

**Published:** 2015-05-26

**Authors:** Yuko Kurushima, Kazunori Ikebe, Ken-ichi Matsuda, Kaori Enoki, Soshiro Ogata, Motozo Yamashita, Shinya Murakami, Yoshinobu Maeda, Osaka Twin Research Group

**Affiliations:** 1 Osaka University Graduate School of Dentistry, Department of Prosthodontics, Gerodontology, and Oral Rehabilitation, Suita, Osaka, Japan; 2 Osaka University Graduate School of Medicine, Center for Twin Research, Suita, Osaka, Japan; 3 Osaka University Graduate School of Dentistry, Department of Periodontology, Suita, Osaka, Japan; University of North Carolina at Chapel Hill, UNITED STATES

## Abstract

**Objective:**

Although researchers have recently demonstrated a relationship between oral health and arterial sclerosis, the genetic contribution to this relationship has been ignored even though genetic factors are expected to have some effect on various diseases. The aim of this study was to evaluate oral health as a significant risk factor related to arterial sclerosis after eliminating genetic confounding through study of older Japanese twins.

**Subjects and Methods:**

Medical and dental surveys were conducted individually for 106 Japanese twin pairs over the age of 50 years. Maximal carotid intima-media thickness (IMT-Cmax) was measured as a surrogate marker of arterial sclerosis. IMT-Cmax > 1.0 mm was diagnosed as arterial sclerosis. All of the twins were examined for the number of remaining teeth, masticatory performance, and periodontal status. We evaluated each measurement related with IMT-Cmax and arterial sclerosis using generalized estimating equations analysis adjusted for potential risk factors. For non-smoking monozygotic twins, a regression analysis using a “between within” model was conducted to evaluate the relationship between IMT-Cmax and the number of teeth as the environmental factor controlling genetic and familial confounding.

**Results:**

We examined 91 monozygotic and 15 dizygotic twin pairs (males: 42, females: 64) with a mean (± standard deviation) age of 67.4 ± 10.0 years. Out of all of the oral health-related measurements collected, only the number of teeth was significantly related to arterial sclerosis (odds ratio: 0.72, 95% confidence interval: 0.52-0.99 per five teeth). Regression analysis showed a significant association between the IMT-Cmax and the number of teeth as an environmental factor (*p* = 0.037).

**Conclusions:**

Analysis of monozygotic twins older than 50 years of age showed that having fewer teeth could be a significant environmental factor related to arterial sclerosis, even after controlling for genetic and familial confounding.

## Introduction

Although tremendous advances have been made in the field of medicine worldwide, vascular diseases are still a major concern. The 2014 Japanese National Population Survey Report found that around one-fourth of all deaths in Japan were due to arteriosclerotic diseases such as cerebrovascular or cardiovascular diseases.

Previous researchers have reported finding a strong association between oral health and arteriosclerotic diseases. Using longitudinal Scottish data, Watt et al. found that while a biological marker of inflammation [C-reactive protein (CRP)] and an indicator of nutritional status [body mass index (BMI)] were not significantly associated with coronary heart diseases, tooth loss was; with edentate subjects having a 2.97 times higher risk for stroke-related mortality [1]. Joshipura et al. and Paunio et al. also suggested that periodontal disease and fewer teeth may be associated with an increased risk of ischemic stroke [2] [3]. These studies were based on a prospective epidemiological aspect and were well designed to investigate which factor significantly affected the outcome within the observed period. It has also been reported that the biological etiology of inflammation involves bacterial infection and that this is a common major factor between tooth loss and vascular diseases [4]. However, no reports have clarified the association between oral health and arteriosclerotic diseases from classical twin design, even though both diseases can be reduced by a modification of both behavior and habits. Moreover, despite the fact that there may be a common genetic intervention, currently no evidence suggests that genetic factors can affect this association. Therefore, further studies that specifically examine the role oral health plays as an environmental factor in arteriosclerotic diseases in the absence of genetic contributions need to be undertaken.

The study of twins is a well-known and unique method that is used to investigate genetic contributions to physical features and diseases in human. The two types of twins are monozygotic (MZ) and dizygotic (DZ) twins, with MZ twins sharing 100% of their genes with each other. Thus, by focusing only on MZ twins when comparing the phenotype between the MZ pairs, it becomes possible to match for genes identical by descent.

The aim of the present study was to investigate the association between arterial sclerosis and oral health, which included factors such as the number of remaining teeth, average probing pocket depth and masticatory performance. To avoid any potential genetic confounding, this study focused on older Japanese MZ twins.

## Materials and Methods

### Study Population

This cross-sectional study enrolled middle-aged and older Japanese twins recruited from all over Japan. The study took place at the Osaka University Center for Twin Research (OUCTR), founded in 2009 [5]. OUCTR is a 4-year project that has been financially supported by the Ministry of Education, Culture, Sports, Science and Technology, Japan (MEXT) since 2011, and MEXT KAKENHI Grant Number 24659857. Eligibility criteria required that the twins had to be of the same sex, be over 20 years of age, and be able to be present at the research center at the same time. In the present study, we only used data for twin pairs that were older than 50 years of age. Written informed consent was obtained from all participants prior to clinical examinations. This study was independently approved by the Osaka University Graduate School of Dentistry Ethics Committee (H21-E2). Every clinical investigation was conducted according to the principles expressed in the Declaration of Helsinki.

Zygosity of the pairs was confirmed using 15 short tandem repeat (STR) markers derived from the blood, as these have previously been shown to be both accurate and reliable [6] [7]. Twin pairs that were completely concordant for these STR markers were designated monozygotic. All other pairs were designated dizygotic.

### Measurements

We evaluated oral health by measuring the number of remaining teeth, masticatory performance, and periodontal status. The number of teeth and masticatory performance were examined by one of two trained and experienced prosthodontists who were blinded to the twin’s zygosity. After being given a piece of gummy jelly (UHA Mikakuto Co., Osaka, Japan), which is a standardized testing food, participants were instructed to chew the piece 30 times on their preferred chewing side and then expectorate the comminuted particles. Masticatory performance was scored using a scale of 0 to 9 by comparing images of visual samples of the particles [8]. To evaluate periodontal status, one of three experienced periodontists recorded probing depth. After assessing the probing depth in six sites (mesiobuccal, mid-buccal, distobuccal, mesiolingual, mid-lingual, and distolingual), the average pocket depth for all of the sites was used for the study analysis.

Increased carotid intima-media thickness (CIMT) has been shown to be a predictor of several kinds of vascular disease [9]. Since an ultrasonic device can be used to perform simple and noninvasive measurements, it is well suited for use in epidemiological studies [10] [11] [12]. In this study, we also used CIMT as a surrogate end point of arterial sclerosis. As CIMT is a measure of the subclinical arterial sclerosis that is correlated with coronary heart disease risk factors, it has been shown to be predictive of subsequent myocardial infarction and stroke [13]. Moreover, it has been suggested that thickening of the common carotid artery might be more representative of the total body arteriosclerotic burden.

At the present time, there is some controversy over whether mean or maximal measurements should be used for the data analysis. Since the maximal CIMT (IMT-Cmax) has been reported to have the highest reliability, validity and reproducibility [14], we used IMT-Cmax score as a continuous variable in our current study. In addition, individuals with IMT-Cmax > 1.0 mm were diagnosed as having arterial sclerosis. This cut-off point was recommended by Japanese guidelines for cervical vessel ultrasound examination.

All relevant data for this study was combined into one dataset (S1 Data).

### Statistical Analysis

Age, sex, BMI, and smoking status are considered to be major risk factors related to both oral health and arterial sclerosis. The conventional tool of risk factor analyses in epidemiology is the regression model, but it is improper to transfer such conventional regression methods to twin data because there is a correlation between twins; the paired structure of the data needs to be taken into account in twin study.

As the first analysis, we evaluated for a univariate correlation between all factors and continuous values of IMT-Cmax using generalized estimating equations (GEE) analysis. The design of GEE is conceptually equivalent to a matched case control design, as it controls the clustering of twins within a pair. Subsequently we evaluated single correlation between all factors and categorical values of arterial sclerosis using the same method, GEE.

In the second step, we used the multivariate GEE method to examine the association between each of the three oral health-related measurements and arterial sclerosis. In this step we adjusted for age, sex, BMI, and smoking status. Only the number of teeth (NT) was a significantly associated with arterial sclerosis within this analysis.

When performing classical twin studies, the assumption is that MZ twins completely share the same genes. Because of this assumption, it is possible to eliminate genetic and family environmental confounding by focusing only on the MZ twins. The paired structure of data from the twins makes it possible to determine if any differences exist. Thus, any variations that may have arisen between the pair can be distinguished by performing regression analysis using a “between within” model [15,16]. This regression analysis allows the covariate effect to differ within and between twin pairs. We formulated regression equations that consisted of the outcome (IMT-Cmax) and the explanatory variables (the twin-pair average of NT and the twin-pair deviation of NT). The within-pair coefficient gives the expected change in IMT-Cmax for a one-tooth change in the difference between the individual NT and the twin-pair average NT value. The between-pair coefficient gives the expected change in the IMT-Cmax for a one-tooth change in the twin-pair average NT. A classical twin study can consider the within-pair coefficient as an environmental effect and can consider the between-pair coefficient as a genetic effect. Based on this regression analysis, we were able to characterize the proportion of the genetic contribution to the association between number of teeth and IMT-Cmax from the coefficient of the twin-pair average NT. In the same way, we were able to characterize the proportion of the environmental contribution to the association between the number of teeth and IMT-Cmax from the coefficient of the twin-pair deviation of the NT. While there were no issues with regard to controlling the age and sex of the subjects analyzed, we only enrolled non-smoking MZ twins in order to ensure that we could also control smoking status of the subjects in this analysis. Statistical analysis was conducted using a “between within” model in the regression analysis with R statistical software version 3.1.2. For each statistical analysis, a *p* value < 0.05 was considered statistically significant.

## Results

Table 1 presents the demographic distribution of all twin pairs over 50 years old. Of the 169 enrolled same-sex twin pairs, 106 were over 50 years of age, with a mean age of 67.4 ± 10.0 years. Out of the 106 twin pairs, 91 were classified as MZ while 15 were DZ. The MZ group consisted of 42 male and 64 female twin pairs, while the DZ group consisted of eight male and seven female twin pairs.

**Table 1:** Participant characteristics.

Variables		Total (n = 212)		MZ (n = 182)		DZ (n = 30)		*P*-value‡
		Mean	SD	Mean	SD	Mean	SD	
Age		67.4	10.0	67.0	10.1	70.1	9.4	0.68
Number of teeth		21.1	9.3	21.1	9.2	20.1	10.0	0.46
BMI (kg/㎡)		22.5	3.1	22.4	3.1	22.8	3.2	0.89
Average pocket depth (mm)		2.8	0.6	2.8	0.6	2.8	0.6	0.31
IMT-Cmax (mm)		0.67	0.24	0.66	0.24	0.71	0.24	0.90
		(n)	Percentage (%)	(n)	Percentage (%)	(n)	Percentage (%)	*P*-value‡
Sex	Male	84	39.6	68	32.1	16	53.3	0.10
	Female	128	60.4	114	67.9	14	46.7	
Smoking status	Never	138	65.1	120	65.9	18	60.0	0.15
	Former	44	20.8	40	22.0	4	13.3	
	Current	30	14.2	22	12.1	8	26.7	
Masticatory performanse score	0~3	44	21.4	33	18.8	11	36.7	0.21
	4~6	101	49.0	91	51.7	10	33.3	
	7~9	61	29.6	52	29.5	9	30.0	
Arterial sclerosis	No	201	94.8	174	96.6	27	90.0	0.20
	Yes	11	5.2	8	4.4	3	10.0	

Continuous variables are presented as mean and SD (Standard Deviation), whereas categorical variables are presented as frequency and percentages.

^‡^, p-value was calculated by Student t-test, Mann-Whitney’s U test, or chi-square test to examine the difference between MZ and DZ

MZ, Monozygotic twins; DZ, Dizygotic twins.

IMT-Cmax, Maximum Carotid intima-media thickness

Table 2 shows the results of the analysis for a single correlation between all factors and IMT-Cmax using GEE. Age, former smoking status, and all the oral health-related measurements were significantly and independently associated with IMT-Cmax. Being older, female, a smoker, and having high BMI, fewer teeth, high score of average periodontal probing pocket, and low score of masticator performance, tended to be negatively related to IMT-Cmax. Table 3 shows the results of the analysis for a single correlation between all factors and arterial sclerosis. The result was the same as that for Table 2. Tooth loss, deep periodontal probing pocket, and low masticatory performance were also significantly related to arterial sclerosis.

**Table 2:** Association between all variables and IMT-Cmax in GEE analysis.

Variables		(n)	Estimate	95% CI
Age		(212)	0.31	(0.17, 0.45)
Sex	Male	(84)		
	Female	(128)	-0.17	(-0.34, 0.0001)
Smoking status	Never	(138)		
	Former	(44)	0.11	(0.04, 0.18)
	Current	(30)	0.09	(-0.02, 0.19)
BMI (kg/㎡)		(212)	-0.06	(-0.32, 0.19)
Number of teeth†		(212)	-0.032	(-0.061, -0.004)
Average pocket depth(mm)		(194)	0.20	(0.02, 0.39)
Masticatory performance score		(206)	-0.04	(-0.08, -0.00002)

CI, Confidence Interval

^†^Number of teeth, 5 teeth for 1 unit

**Table 3:** Association between all variables and arterial sclerosis in GEE analysis.

Variables		(n)	Odds rate	95% CI
Age		(212)	1.09	(1.02, 1.17)
Sex	Male	(84)		
	Female	(128)	0.53	(0.13,2.16)
Smoking status	Never	(138)		
	Former	(44)	4.48	(1.12, 17.96)
	Current	(30)	2.60	(0.37, 18.22)
BMI (kg/㎡)		(212)	1.12	(0.90, 1.40)
Number of teeth†		(212)	0.65	(0.51, 0.83)
Average pocket depth(mm)		(194)	2.98	(1.34, 6.59)
Masticatory performance score		(206)	0.74	(0.60, 0.91)

CI, Confidence Interval

^†^Number of teeth, 5 teeth for 1 unit

Table 4 shows the results of the association between each oral health-related measurement and IMT-Cmax after adjusting for age, sex, BMI, and smoking status as potential risk factors. No significant differences existed between the three oral health-related measurements and IMT-Cmax. Table 5 shows the results of the association between each oral health-related measurement and arterial sclerosis after adjusting for potential risk factors. The only factor significantly related to arterial sclerosis was the number of teeth. No significant relationship was found either for the masticatory performance or for the average probing pocket depth with regard to arterial sclerosis. Our results also showed that if a person had five more teeth than another person, the prevalence ratio of arterial sclerosis was 28% less for the person with the larger number of teeth.

**Table 4:** Association between each oral health-related measurement and IMT-Cmax after adjusting for age, sex, smoking status, and BMI using GEE analysis.

Variables		Estimate	95% CI	Estimate	95% CI	Estimate	95% CI
Age		0.005	(-0.001, 0.010)	0.005	(0.001, 0.010)	0.007	(0.003, 0.010)
Sex	Male						
	Female	-0.02	(-0.13, 0.10)	-0.016	(-0.121, 0.090)	-0.016	(-0.125, 0.094)
Smoking status	Never						
	Former	0.07	(-0.01, 0.14)	0.065	(-0.001, 0.138)	0.076	(0.007, 0.144)
	Current	0.06	(-0.02, 0.14)	0.11	(0.001, 0.21)	0.074	(-0.010, 0.158)
BMI (kg/㎡)		-0.006	(-0.026, 0.013)	-0.005	(-0.025, 0.015)	-0.007	(-0.027, 0.014)
Number of teeth*		-0.02	(-0.37, 1.50)	-	-	-	-
Average pocket depth(mm)		-	-	0.05	(-0.03, 0.13)	-	-
Masticatory performance score		-	-	-	-	-0.021	(-0.067, 0.025)

CI, Confidence Interval

*Number of teeth, 5 teeth for 1 unit

**Table 5:** Association between each oral health-related measurement and arterial sclerosis after adjusting for age, sex, smoking status, and BMI using GEE analysis.

Variables		Odds rate	95% CI	Odds rate	95% CI	Odds rate	95% CI
Age		1.02	(0.95, 1.10)	1.09	(1.004, 1.19)	1.05	(0.98, 1.12)
Sex	Male						
	Female	1.19	(0.19, 7.44)	1.84	(-0.37, 9.21)	1.72	(0.34, 8.71)
Smoking status	Never						
	Former	3.00	(0.85, 10.55)	4.27	(1.49, 12.29)	3.23	(0.90, 11.62)
	Current	2.14	(0.21, 22.17)	6.78	(0.81, 56.55)	2.21	(0.23, 21.50)
BMI (kg/㎡)		0.99	(0.77, 1.26)	1.11	(0.86, 1.45)	1.10	(0.84, 1.45)
Number of teeth*		0.72	(0.52, 0.99)	-	-	-	-
Average pocket depth(mm)		-	-	2.54	(0.83, 7.77)	-	-
Masticatory performance score		-	-	-	-	0.84	(0.62, 1.13)

CI, Confidence Interval

*Number of teeth, 5 teeth for 1 unit

Overall, the “between within” model in regression analysis showed that the deviation of the number of teeth was significantly related to IMT-Cmax (*p* = 0.037) (Table 6) and indicated that the number of teeth was significantly related with IMT-Cmax as an environmental factor controlled by age, sex, and genetic and family environmental factors.

**Table 6:** Association between the number of teeth and IMT-Cmax in regression analysis using the “between within” model.

	Estimate	SE	t-value	*p*-value
Average of the number of teeth between the pair(Genetic and familial effect)	-0.48	0.26	-1.85	0.064
Deviation of the number of teeth between the pair(Environmental effect)	-0.85	0.30	-2.88	0.037

## Discussion

Our current results indicate that the number of teeth is an important environmental factor related to arterial sclerosis, even after taking the genetic contribution into consideration. To the best of our knowledge, this is the first study that has investigated the association between oral health and arterial sclerosis while considering genetic confounding.

However, many studies have reported findings that both tooth loss and arterial sclerosis can be attributed, to some degree, to genetic factors. The findings of a large Swedish survey performed by Mucci et al. implied that genetic factors contributed to 14% of the variation in tooth loss among women and to 39% among men [17]. The heritability of arteriosclerotic diseases was found to be comparatively high, ranging between approximately 35% [18] [19] to 65% [20]. Song et al. further reported that genetic factors may play an important role in the development of coronary heart diseases, even in the absence of environmental factors [21]. These previous reports have clarified that genetic factors related to both arteriosclerotic diseases and oral health can act independently. Based on all of these previous findings, we suggest that there might be a common genetic factor associated with both oral health and arterial sclerosis.

Beck et al. also hypothesized that there are some genes responsible for regulating the T-cell monocyte response and the host-microbial environment, which can spark and modulate the response, although this hypothesis has yet to be confirmed [22].

Our results imply that the number of teeth might be the most appropriate and comprehensive indicator of oral health, which is consistent with findings from previous studies [23] [24]. It has been reported that advanced dental caries and fatal periodontal disease lead to tooth loss, which can attenuate masticatory ability and oral function [25] [26]. Furthermore, tooth loss is considered to degrade nutritional intake, even after prosthodontic treatments [27] [28]. As compared to full-dentate subjects, people who lack teeth may be at a nutritional disadvantage with regard to the intake of fruits, vegetables, beta carotene, folate, and vitamin C [29]. Although we did not identify the biological etiology, tooth loss could be comprehensively related to arterial sclerosis. In past studies, some evidence of the etiology of this relationship has been reported. Periodontal disease, which is the major cause of tooth loss, causes inflammation due to bacterial infection and leads to an increase in the IMT-Cmax [30] [31]. However, we believe that these results also suggest that tooth loss is a cause of the increased IMT-Cmax.

One of the important points of the current study was the method of our data sampling, as each medical and dental specialist collected several kinds of clinical measurements from each of the individuals. Thus, the data collected in the current study are more reliable than the data obtained from previous twin studies by questionnaire or telephone interview.

In contrast to the high reliability of the data itself, the sample size of our data was small. Due to this small sample size, we were not able to conduct a multivariate difference regression analysis with a large number of clinical measurements for the explanatory variable. If more measurements related to arterial sclerosis can be obtained and used as explanatory variables for a multivariate analysis, this could lead to a more in-depth examination of the confounding effects of other potential factors. Greater amounts of reliable data would also make it possible to fully exploit the strength of our study. Although we could not adjust for several of the measurements related to arterial sclerosis, it was interesting that even only a small twin sample was able to generate a significant association between tooth loss and IMT-Cmax.

In the current study, we only analyzed samples from subjects who were over 50 years age, even though this resulted in a smaller sample size. The reason we chose this age group was that there is a distinct difference in the variability in the number of teeth between subjects under and over 50 years of age (*p* value of Mann-Whitney U test: < 0.001). The age of 50 years is usually considered to be the point where various kinds of chronic diseases, involving both oral health and vascular diseases, are more commonly observed.

Regarding methodology, twin studies are unique in that it is possible to detect genetic and environmental factors when using quantitative genetic analysis. Co-twin control analyses, as well as regression analyses that use the “between within” model, are able to detect genetic contributions and the association of several individual measurements. It is well known that classical twin analysis can be used to compare the odds ratio of GEE in all twins, while the odds ratio of a co-twin control analysis can only be performed in discordant MZ twins or in discordant DZ twins [32]. With regard to the resources available to us in the current study, we felt our approach using a regression analysis would provide better results than that which could be obtained when using a co-twin control analysis. Furthermore, the limited number of patients in our sample group meant the prevalence of arterial sclerosis was quite small, and thus, while GEE can be performed with such small sample sizes; it is not possible for a co-twin control analysis.

While our present findings provide the basis for further analyses designed to more specifically investigate these potential relationships, our results also appear to infer that tooth loss is a significant environmental factor that could affect arterial sclerosis.

## Supporting Information

S1 Data Relevant data needed for all the analyses in this study. (CSV)
